# Association of Maternal Depressive Symptoms During the Perinatal Period With Oppositional Defiant Disorder in Children and Adolescents

**DOI:** 10.1001/jamanetworkopen.2021.25854

**Published:** 2021-09-30

**Authors:** Berihun Assefa Dachew, James G. Scott, Jon E. Heron, Getinet Ayano, Rosa Alati

**Affiliations:** 1 School of Population Health, Curtin University, Perth, Australia; 2 QIMR Berghofer Medical Research Institute, Herston, Australia; 3 Queensland Centre for Mental Health Research, The Park Centre for Mental Health, Brisbane, Australia; 4 Metro North Mental Health, Royal Brisbane and Women’s Hospital, Herston, Australia; 5 Department of Population Health Sciences, University of Bristol, Bristol, United Kingdom; 6 Research Training Department, Amanuel Mental Specialized Hospital, Addis Ababa, Ethiopia; 7 Institute for Social Science Research, University of Queensland, Brisbane, Australia

## Abstract

**Importance:**

An association between perinatal maternal depression and risk of oppositional defiant disorder (ODD) in offspring has not been established. Identifying early determinants of ODD can help inform preventative intervention efforts.

**Objective:**

To investigate the association between maternal perinatal depressive symptoms and the risk of ODD in offspring aged 7 to 15 years.

**Design, Setting, and Participants:**

This population-based longitudinal birth cohort study used data from the Avon Longitudinal Study of Parents and Children (ALSPAC), in Bristol, UK. All pregnant women residents in Avon, UK, with expected delivery dates from April 1, 1991, to December 31, 1992, were invited to participate in the study. The study cohort ranged from approximately 8000 (at 7 years of age) to 4000 (at 15 years of age) mother-offspring pairs. Data were analyzed from November 2020 to July 2021.

**Main Outcomes and Measures:**

Maternal depressive symptoms were measured using the Edinburgh Postnatal Depression Scale (EPDS) antenatally at 18 and 32 weeks of gestation and postnatally at 8 weeks and 8 months. This study primarily used a cutoff score of 12 or more on the EPDS to identify mothers with symptoms of depression, and the continuous EPDS scores were used to confirm the results of the main analyses. Offspring ODD at 7, 10, 13, and 15 years of age were diagnosed using the parent-reported Development and Well-Being Assessment.

**Results:**

Of 7994 mother-offspring pairs for whom data were available on offspring ODD at 7 years, 4102 offspring (51.3%) were boys. The mean (SD) age of mothers was 28.6 (4.6) years. Maternal antenatal depressive symptoms (measured at 32 weeks of gestation) were associated with offspring ODD (adjusted odds ratio [AOR], 1.75; 95% CI, 1.33-2.31). Offspring of mothers with postpartum depressive symptoms at 8 weeks and 8 months were more than 2 times more likely to have a diagnosis of ODD over time (AOR at 8 weeks, 2.24 [95% CI, 1.74-2.90]; AOR at 8 months, 2.04 [95% CI, 1.55-2.68]), and maternal persistent depressive symptoms were associated with a 4-fold increased risk of offspring ODD (AOR, 3.59; 95% CI, 1.98-6.52).

**Conclusions and Relevance:**

These findings suggest that perinatal depressive symptoms are associated with ODD in offspring and further support the need for early identification and management of prenatal and postnatal depression in women of childbearing age.

## Key Points

**Question:**

Are maternal perinatal depressive symptoms associated with an increased risk of oppositional defiant disorder (ODD) in children and adolescents?

**Findings:**

In this population-based birth cohort study of 7994 mother-offspring pairs, maternal perinatal depressive symptoms were associated with offspring ODD. Persistent depressive symptoms in mothers during pregnancy and the postpartum period were associated with a 4-fold increased risk of ODD over time.

**Meaning:**

These findings suggest that identifying and treating maternal mental health problems during the perinatal period, especially among those who have postnatal and/or persistent depressive symptoms, may reduce the risk of ODD in children and adolescents.

## Teaser

This population-based cohort study investigates the association between maternal antenatal and postnatal depressive symptoms and the risk of oppositional defiant disorder in offspring aged 7 to 15 years.

## Introduction

Oppositional defiant disorder (ODD) is a disruptive behavior disorder characterized by a persistent pattern of angry/irritable mood, argumentative/defiant behavior, and/or vindictiveness^1^ with a prevalence in children of 3.6% (95% CI, 2.8%-4.7%).^2^ Symptoms generally begin during the preschool years, with diagnosis typically occurring during middle childhood (6 to 13 years of age).^3,4^ Before adolescence, ODD is more prevalent in boys than in girls, with a ratio of male to female prevalence of 1.4:1.^1^

Children with ODD have difficulty functioning at school and home and are at increased risk of other comorbid disorders, including conduct disorder, attention-deficit/hyperactivity disorder (ADHD), and mood, anxiety, and substance use disorders.^5,6^ Identifying early-life risk factors of ODD is, therefore, important to achieve a better understanding of the etiology of the disorder and ultimately devise targeted interventions for those affected.^7^

Both genetic and environmental risk factors are involved in the development of behavioral disorders in children and adolescents, including ODD.^8,9,10,11^ Maternal mental health problems during the perinatal period are among these factors.^11,12,13,14,15^ Most of the studies that have examined the effects of maternal perinatal depression on behavioral and emotional problems in offspring have reported positive associations.^12,13,14,15,16^ For example, a recent meta-analysis^12^ found that maternal perinatal depression (both during and after pregnancy) were associated with adverse socioemotional, cognitive, and adaptive behavior development in offspring. A prospective cohort study^15^ based on 2 pregnancy cohorts, the Avon Longitudinal Study of Parents and Children (ALSPAC) and the Generation R Study, also reported positive associations between maternal prenatal depressive symptoms and attention problems in offspring in both cohorts (odds ratio [OR] for ALSPAC, 1.33 [95% CI, 1.19-1.48]; OR for Generation R, 1.23 [95% CI, 1.05-1.43]).

To our knowledge, no previous studies have examined the association between maternal depressive symptoms during the perinatal period and the risk of ODD in children and adolescents. In the present study, our primary aim was to investigate the association between maternal antenatal and postnatal depressive symptoms and the risk of ODD in offspring throughout childhood and adolescence. We used data from ALSPAC, a birth cohort study with rich data that allowed us to observe ODD risk throughout childhood and adolescence. The secondary objective was to investigate the association between persistent depressive symptoms in mothers and ODD in offspring over time. We achieved this by comparing offspring of mothers who had depressive symptoms during the antenatal and postnatal periods with offspring of mothers with no depressive symptoms. We hypothesized that the association between perinatal depressive systems and offspring ODD would be stronger for offspring of mothers who had both antenatal and postnatal depressive symptoms. We then examined these associations at 7, 10, 13, and 15 years of age.

## Methods

### Study Design and Participants

In this cohort study, we used data from the ALSPAC, an ongoing population-based longitudinal birth cohort in Bristol, UK.^17,18,19^ The details of the cohort, including the participants for the present study, are found in the eMethods, the eFigure, and eTable 1 in the Supplement. All pregnant women residents in Avon, UK, with expected delivery dates from April 1, 1991, to December 31, 1992, were invited to participate in the study. Ethical approval for the ALSPAC study was obtained from the ALSPAC Ethics and Law Committee as well as the local research ethics committees. Written informed consent for the use of data collected via questionnaires and clinics was obtained from participants following the recommendations of the ALSPAC Ethics and Law Committee at the time. The study website includes information on ethical approval, including the dates of approval and associated reference numbers.^20^ This study followed the Strengthening the Reporting of Observational Studies in Epidemiology (STROBE) reporting guideline.

### Measures

#### Exposures

Maternal depressive symptoms were measured using the Edinburgh Postnatal Depression Scale (EPDS). The EPDS is a widely used 10-item self-report depression questionnaire that has been shown to be valid within and outside the postnatal period.^21^ Possible scores range from 0 to 30, with higher scores indicating greater severity of depressive symptoms. Scores of 12 or more have high specificity and sensitivity for clinically diagnosed depressive disorders.^21,22^ Participants completed the EPDS at 18 and 32 weeks antenatally and at 8 weeks and 8 months postnatally. This study primarily used a cutoff score of 12 or more to identify mothers who were experiencing symptoms of antenatal and postnatal depression, and the continuous EPDS scores were used to confirm the results of the main analyses.

#### Outcome

Oppositional defiant disorder in offspring at 7, 10, 13, and 15 years of age was assessed by using parental reports of the Development and Well-Being Assessment (DAWBA). The DAWBA is a validated diagnostic instrument combining structured and semistructured questions that establish the presence of child and adolescent mental health disorders.^23^ Prompts are consistent with the *Diagnostic and Statistical Manual of Mental Disorders, 4th edition,* and the* International Statistical Classification of Diseases and Related Health Problems, Tenth Revision*.^23^ Responses are entered into a computer program, and likely diagnoses are assessed by experienced clinical raters who decide whether to accept or overturn the computer diagnosis. Validation studies show substantial agreement between diagnoses generated by the DAWBA and clinician diagnoses, with κ coefficients of 0.83 (95% CI, 0.68-0.97) for any disorder, 0.84 (95% CI, 0.69-0.99) for any internalizing disorder, 0.89 (95% CI, 0.77-1.00) for any externalizing disorder, and 0.79 (95% CI, 0.39-1.00) for any other disorder.^24^ Conduct disorder, ADHD, and depression in offspring at 7, 10, 13, and 15 years of age were also measured using the DAWBA.

### Confounders

Data on potential confounders were obtained from obstetric records and questionnaires administered during pregnancy. These included maternal age at conception, maternal educational attainment, ethnicity, parity, maternal prepregnancy body mass index, tobacco use (smoking) during pregnancy, diabetes status during pregnancy, urinary tract infections during pregnancy, preeclampsia, alcohol consumption during pregnancy, and prenatal anxiety and offspring sex and gestational age at delivery. Covariates were selected based on previous reports of their association with maternal perinatal depression and offspring behavioral and emotional problems.^25,26,27,28,29^

### Statistical Analysis

Data were analyzed from November 2020 to July 2021. First, we compared participants with and without a diagnosis of ODD on the key sociodemographic and clinical characteristics using cross-tabulations and χ^2^ test statistics. Then, we conducted a series of generalized estimating equation (GEE) models to investigate the association between maternal antenatal and postnatal depressive symptoms and ODD in offspring across the 4 measurement periods (ie, 7, 10, 13, and 15 years of age), computing ORs and 95% CIs as a measure of risk. The GEE model accounts for correlation due to repeated measures being included at the 4 different periods.^30^ Model 1 included univariable associations between maternal perinatal depression and ODD in offspring over time. Model 2 was adjusted for sociodemographic, health, and behavioral factors. Model 3 was adjusted for all confounders previously included in model 2, plus comorbid conduct disorder, and model 4 was further adjusted for comorbid ADHD. These analyses were performed for each of the 2 antenatal (18 and 32 weeks) and postnatal (8 weeks and 8 months) EPDS measures separately. We also examined the effects of persistent depressive symptoms during the perinatal period on ODD in offspring by comparing offspring of mothers who had depressive symptoms in all 4 measurement periods (ie, at 18 and 32 weeks of gestation and postnatal 8 weeks and 8 months) with those of offspring of mothers with no depressive symptoms.

We used separate logistic regression analyses to further examine the association between maternal depressive symptoms during pregnancy and the postpartum period and ODD in offspring at each developmental period (ie, 7, 10, 13, and 15 years of age). The models described above (in GEE models) were repeated using logistic regression analyses.

To account for missing data, we conducted multiple imputations.^31^ We used 100 cycles of regression switching and generated 100 imputed data sets. All outcome data and covariates included in the regression model and additional auxiliary variables associated with incomplete variables were imputed, and the analyses were repeated. Statistical analyses were conducted using STATA software, release 16 (StataCorp LLC).^32^ All statistical tests were conducted with 2-tailed statistical significance levels set at *P* ≤ .05.

## Results

Table 1 summarizes the sociodemographic, clinical, and behavioral characteristics of study participants. Of 7994 mother-offspring pairs for whom data were available on ODD in offspring at 7 years, 4102 offspring (51.3%) were boys and 3892 (48.7%) were girls. The mean (SD) age of mothers at delivery was 28.6 (4.6) years. A total of 1511 of 7830 mothers (19.3%) smoked tobacco and 1196 of 7790 (15.4%) consumed alcohol during their pregnancy. The prevalence of antenatal depressive symptoms was 1072 of 7261 (14.8%) at 18 weeks of gestation and 1285 of 7505 (17.1%) at 32 weeks of gestation, and 890 of 7563 mothers (11.8%) had postnatal depressive symptoms at 8 weeks.

**Table 1.** Sociodemographic, Behavioral, and Clinical Characteristics of Study Participants Who Had Complete Data on ODD Diagnosis at 7 Years of Age

Characteristic	Data^a^
Maternal age at delivery, mean (SD), y (n = 7967)	28.6 (4.6)
Maternal educational level	
CSE	822 (11.0)
Vocational^b^	685 (9.2)
O level	2726 (36.6)
A level	1985 (26.6)
Degree	1232 (16.5)
Parity	
Nullipara	3574 (46.3)
Multipara	4153 (53.7)
Prepregnancy BMI	
<18.5	327 (4.5)
18.5-24.9	5509 (75.8)
≥25.0	1430 (19.7)
Hypertensive disorders during pregnancy	
Yes	6675 (84.2)
No	1257 (15.8)
Pregnancy diabetes status	
Glycosuria or diabetes (existing/gestational)	317 (4.1)
No glycosuria or diabetes	7397 (95.9)
UTI during pregnancy	
Yes	410 (5.3)
No	7155 (94.6)
Alcohol consumption in pregnancy	
Yes	1196 (15.4)
No	6594 (84.6)
Smoking during pregnancy	
Yes	1511 (19.3)
No	6319 (80.7)
Maternal antenatal anxiety symptoms	
Yes	1509 (20.5)
No	5835 (79.5)
Maternal antenatal depressive symptoms at 18 wks	
Yes	1072 (14.8)
No	6189 (85.2)
Maternal antenatal depressive symptoms at 32 wk	
Yes	1285 (17.1)
No	6220 (82.9)
Maternal postnatal depressive symptoms at 8 wk	
Yes	890 (11.8)
No	6673 (88.2)
Maternal postnatal depressive symptoms at 8 mo	
Yes	763 (10.2)
No	6728 (89.8)
Sex of offspring	
Male	4102 (51.3)
Female	3892 (48.7)
Gestational age at delivery, wk	
<37	340 (4.3)
≥37	7627 (95.7)

Abbreviations: BMI, body mass index (calculated as weight in kilograms divided by height in meters squared); CSE, Certificate of Secondary Education; ODD, oppositional defiant disorder; UTI, urinary tract infection.

^a^ Unless otherwise indicated, data are expressed as number (%) of offspring. Percentages have been rounded and may not total 100.

^b^ O level indicates examinations taken and passed at 16 years of age; A level, examinations taken and passed at 18 years of age on leaving secondary school.

Table 2 compares offspring with and without a diagnosis of ODD across the 4 measurement periods (7, 10, 13, and 15 years of age) by the key sociodemographic and clinical characteristics. Mothers of children with ODD were more likely to smoke during pregnancy and report anxiety and depressive symptoms compared with mothers of children without ODD (Table 2). The number and percentage of missing values across the outcomes, exposures, and major covariates are described in eTable 2 in the Supplement. We also compared characteristics of mothers and children with and without data on ODD. Participants with missing data were younger at childbirth, less educated, and more likely to be overweight/obese, to have urinary tract infections during pregnancy, to smoke tobacco, and to have higher levels of anxiety and depressive symptoms (eTable 3 in the Supplement).

**Table 2.** Characteristics of Study Participants Included in the Analyses by ODD Diagnosis^a^

Characteristic	Offspring age											
	7 y^b^			10 y^c^			13 y^d^			15 y^e^		
	With ODD	Without ODD	*P* value^f^	With ODD	Without ODD	*P* value^f^	With ODD	Without ODD	*P* value^f^	With ODD	Without ODD	*P* value^f^
Maternal age ≥35 y	32 (11.7)	915 (11.9)	.09	28 (11.5)	850 (12.1)	.45	20 (9.3)	805 (12.6)	<.001	11 (6.9)	560 (13.7)	.09
Maternal educational level CSE	31 (12.4)	791 (11.0)	.52	28 (12.3)	686 (10.4)	.74	38 (19.9)	569 (9.5)	<.001	18 (12.2)	316 (7.9)	.28
Nullipara	115 (43.6)	3459 (46.4)	.37	116 (48.7)	3161 (46.4)	.47	90 (43.1)	2957 (47.6)	.20	71 (47.0)	2042 (49.4)	.56
Prepregnancy BMI ≥25.0	46 (19.1)	1384 (19.7)	.93	41 (18.7)	1252 (19.6)	.09	44 (22.7)	1125 (19.2)	.26	34 (23.6)	736 (18.9)	.34
Hypertensive disorders of pregnancy	53 (19.5)	1204 (15.7)	.10	30 (12.5)	1110 (15.9)	.15	33 (15.5)	1030 (16.2)	.77	16 (10.2)	699 (16.5)	.04
Pregnancy diabetes	22 (8.5)	295 (4.0)	<.001	9 (3.9)	268 (3.9)	.98	6 (2.9)	246 (4.0)	.42	7 (4.6)	165 (4.0)	.70
UTI during pregnancy	20 (7.9)	390 (5.3)	.08	15 (6.5)	351 (5.3)	.41	15 (7.3)	312 (5.1)	.18	7 (4.6)	228 (5.62)	.59
Alcohol consumption during pregnancy	56 (21.1)	1140 (15.2)	.01	47 (19.7)	1037 (15.1)	.05	39 (18.8)	944 (15.1)	.15	24 (15.9)	627 (15.0)	.77
Smoking during pregnancy	86 (32.2)	1425 (18.8)	<.001	66 (27.6)	1232 (17.9)	<.001	60 (28.7)	1076 (17.1)	<.001	34 (22.2)	616 (14.7)	.01
Maternal antenatal anxiety symptoms	96 (38.3)	1413 (19.9)	<.001	77 (34.1)	1246 (19.2)	<.001	62 (31.0)	1142 (19.3)	<.001	39 (26.7)	743 (18.8)	.02
Maternal antenatal depressive symptoms at 18 wk	63 (26.0)	1009 (14.4)	<.001	54 (25.2)	893 (13.9)	<.001	51 (26.3)	830 (14.2)	<.001	30 (21.4)	528 (13.5)	.01
Maternal antenatal depressive symptoms at 32 wk	81 (32.1)	1204 (16.6)	<.001	77 (33.6)	1057 (16.0)	<.001	64 (30.9)	956 (15.8)	<.001	40 (26.3)	619 (15.4)	<.001
Maternal postnatal depressive symptoms at 8 wk	77 (29.7)	813 (11.1)	<.001	55 (24.0)	739 (11.1)	<.001	49 (23.4)	661 (10.9)	<.001	27 (27.9)	1426 (10.6)	.004
Maternal postnatal depressive symptoms at 8 mo	64 (25.9)	699 (9.7)	<.001	57 (25.0)	629 (9.5)	<.001	42 (20.8)	580 (9.6)	<.001	32 (21.6)	362 (9.1)	<.001
Male offspring	205 (74.3)	3897 (50.5)	<.001	179 (71.3)	3644 (49.7)	<.001	132 (57.6)	3323 (49.9)	.02	87 (50.9)	2128 (47.8)	.42
Gestational age at delivery <37 wk	19 (7.0)	321 (4.2)	.03	15 (6.2)	297 (4.2)	.14	12 (5.6)	259 (4.1)	.27	10 (6.3)	180 (4.2)	.22

Abbreviations: BMI, body mass index (calculated as weight in kilograms divided by height in square meters); CSE, Certificate of Secondary Education; ODD, oppositional defiant disorder; UTI, urinary tract infection.

^a^ Data are expressed as number (%) of offspring.

^b^ Sample sizes range from 7261 to 7994 with data available.

^c^ Sample sizes range from 6608 to 7586 with data available.

^d^ Sample sizes range from 6046 to 6884 with data available.

^e^ Sample sizes range from 4031 to 4628 with data available.

^f^ Calculated using the Pearson χ^2^ test.

Data on parental reports of ODD at 7 years of age were available in 7988 offspring; at 10 years of age, in 7588 offspring; at 13 years of age, in 6886 offspring; and at 15 years of age, in 4630 offspring. The prevalence of ODD in offspring was 276 (3.5%) at 7 years of age, 251 (3.3%) at 10 years of age, 229 (3.3%) at 13 years of age, and 171 (3.7%) at 15 years of age. The prevalence of ODD slightly decreased in boys from 7 (205 [5.0%]) to 15 (83 [3.9%]) years of age but increased over time in girls (from 71 [1.8%] to 84 [3.5%]) (Figure). Overall, more boys than girls had an ODD diagnosis (χ^2^ = 84.9; *P* < .001). However, no evidence of interaction between antenatal or postnatal maternal depressive symptoms and sex for offspring ODD was found at any age group (odds ratio [OR], < 1.76; *P* > .05 for interaction).

**Figure.**
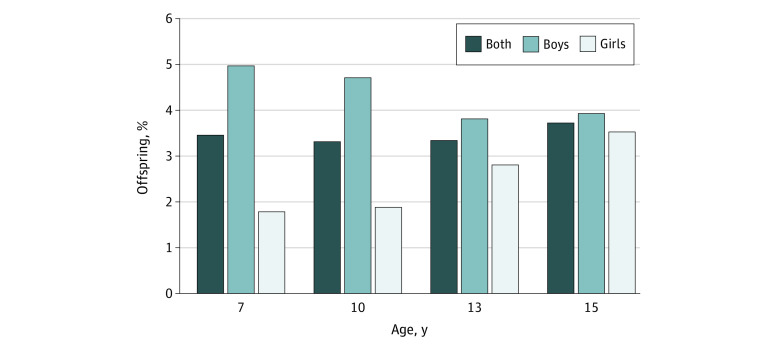
Prevalence of Oppositional Defiant Disorder in Children and Adolescents

Table 3 shows associations between timing of maternal antenatal and postnatal depressive symptoms and ODD in offspring across the 4 measurement periods, before and after adjustment for confounders. After accounting for a wide range of confounders, the results of multivariable GEE analysis (model 2) showed that antenatal depressive symptoms, measured at 18 and 32 weeks of gestation, were associated with a 42% (adjusted OR [AOR], 1.42; 95% CI, 1.09-1.85) and a 75% (AOR, 1.75; 95% CI, 1.33-2.31) increased risk of ODD in offspring over time, respectively. We also found evidence that the offspring of mothers with postpartum depressive symptoms measured at 8 weeks and 8 months were more than 2 times more likely to have a diagnosis of ODD over time (AOR at 8 weeks, 2.24 [95% CI, 1.74-2.90]; AOR at 8 months, 2.04 [95% CI, 1.55-2.68]). Adjustment for antenatal depressive symptoms did not alter these associations (AOR at 8 weeks, 2.12 [95% CI, 1.64-2.74]; AOR at 8 months, 2.04 [95% CI, 1.56-2.67]). The association between maternal third-trimester depressive symptoms measured at 32 weeks of gestation and postnatal depressive symptoms at 8 weeks and 18 months and ODD in offspring remain the same after further adjustment for comorbid conduct disorder and ADHD over time. However, no association between antenatal maternal depressive symptoms at 18 weeks of gestation and ODD in offspring was observed. Further adjustment for comorbid depression in offspring did not alter the findings (eTable 4 in the Supplement). The results were broadly comparable when we repeated the analysis using the continuous EPDS scores (eTable 5 in the Supplement).

**Table 3.** Maternal Antenatal and Postnatal Depressive Symptoms and Risk of ODD in Offspring Over Time in GEE Models

Time of depressive symptoms	No. of observations^a^	Model 1		Model 2		Model 3		Model 4	
		OR (95% CI)^b^	*P* value	OR (95% CI)^c^	*P* value	OR (95% CI)^d^	*P* value	OR (95% CI)^e^	*P* value
Antenatal									
18 wk	20 028	2.09 (1.66-2.64)	<.001	1.42 (1.09-1.85)	.009	1.29 (0.98-1.69)	.07	1.25 (0.94-1.65)	.13
32 wk	20 947	2.40 (1.94-2.95)	<.001	1.75 (1.33-2.31)	<.001	1.75 (1.32-2.31)	<.001	1.43 (1.07-1.92)	.02
Postnatal									
8 wk	20 372	2.94 (2.35-3.68)	<.001	2.27 (1.77-2.90)	<.001	2.24 (1.74-2.90)	<.001	1.87 (1.42-2.45)	<.001
8 mo	20 160	2.78 (2.19-3.54)	<.001	2.18 (1.68-2.83)	<.001	2.04 (1.55-2.68)	<.001	1.93 (1.45-2.56)	<.001

Abbreviations: GEE, Generalized Estimating Equation; ODD, oppositional defiant disorder; OR, odds ratio.

^a^ Indicates the sum of the samples included at each age group (4 measurement periods). For example, for antenatal depressive symptoms measured at a gestational age of 18 weeks, a total of 5991 participants were included at 7 years of age; 5222, at 10 years of age; 5008, at 13 years of age; and 3427, at 15 years of age (see eFigure in the Supplement for details).

^b^ Unadjusted.

^c^ Adjusted for maternal age, income, educational status, ethnicity, parity, prepregnancy body mass index, pregnancy diabetes, urinary tract infection during pregnancy, preeclampsia, alcohol consumption during pregnancy, smoking during pregnancy, and anxiety and offspring sex and gestational age at delivery.

^d^ Adjusted for covariates in model 2 and comorbid conduct disorder over time.

^e^ Adjusted for covariates in model 2 and comorbid conduct and attention-deficit/hyperactivity disorder over time.

We further examined the association between persistent perinatal depressive symptoms and the risk of ODD in offspring by comparing offspring of mothers who had depressive symptoms at the 4 points of measurement (221 [3.4%]) with offspring of mothers with no depressive symptoms (n = 6277). After accounting for a range of known confounders, we found that maternal persistent depressive symptoms were associated with a 4-fold increased risk of offspring ODD over time (AOR, 3.59; 95% CI, 1.98-6.52; *P* < .001) (eTable 6 in the Supplement).

We also ran sensitivity analyses to examine the associations at each measurement period (at 7, 10, 13, and 15 years of age) and found consistent results (Table 4). After adjustments were made for a wide range of confounders in model 2, we found that third-trimester maternal depressive symptoms (measured at 32 weeks of gestation) and postpartum depressive symptoms (measured at 8 weeks and 8 months) were associated with increased risk of ODD across all offspring age groups (AORs, 1.58 [95% CI, 1.06-2.34] to 2.85 [95% CI, 2.01-4.03]). Adjustment for comorbid conduct disorder produced similar results. Some of these associations were, however, attenuated after adjustment for comorbid ADHD. Similar to the GEE models, we found no evidence of association between antenatal depressive symptoms measured at 18 weeks of gestation and ODD in offspring in all age groups. Broadly consistent results were obtained when we reran the analyses using the continuous EPDS scores (eTable 7 in the Supplement) and on the imputed data sets (eTable 8 and eTable 9 in the Supplement).

**Table 4.** Maternal Antenatal and Postnatal Depressive Symptoms and the Risk of ODD in Offspring at Each Time Points Using Logistic Regression Analysis

Time of depressive symptoms	No. of offspring	Model 1		Model 2		Model 3		Model 4	
		OR (95% CI)^a^	*P* value	OR (95% CI)^b^	*P* value	OR (95% CI)^c^	*P* value	OR (95% CI)^d^	*P* value
**Offspring aged 7 y**									
Antenatal									
18 wk	5991	2.11 (1.51-2.94)	<.001	1.36 (0.93-1.99)	.12	1.21 (0.80-1.83)	.36	1.10 (0.70-1.75)	.67
32 wk	6276	2.38 (1.76-3.22)	<.001	1.58 (1.06-2.34)	.02	1.61 (1.06-2.44)	.03	1.18 (0.73-1.90)	.51
Postnatal									
8 wk	6093	3.67 (2.71-5.02)	<.001	2.85 (2.01-4.03)	<.001	2.77 (1.91-4.01)	<.001	2.38 (1.55-3.64)	<.001
8 mo	6041	3.30 (2.36-4.61)	<.001	2.50 (1.73-3.63)	<.001	2.48 (1.66-3.68)	<.001	2.20 (1.40-3.47)	.001
**Offspring aged 10 y**									
Antenatal									
18 wk	5522	2.25 (1.59-3.19)	<.001	1.49 (0.99-2.23)	.003	1.25 (0.80-1.96)	.32	1.24 (0.75-2.05)	.14
32 wk	5778	2.58 (1.88-3.53)	<.001	1.86 (1.23-2.82)	<.001	1.61 (1.02-2.53)	.04	1.49 (0.90-2.45)	.12
Postnatal									
8 wk	5621	2.69 (1.90-3.81)	<.001	2.03 (1.39-2.98)	<.001	1.91 (1.25-2.91)	.003	1.56 (0.96-2.52)	.07
8 mo	5570	3.23 (2.78-4.60)	<.001	2.52 (1.71-3.71)	<.001	2.27 (1.49-3.48)	<.001	2.12 (1.32-3.42)	.002
**Offspring aged 13 y**									
Antenatal									
18 wk	5088	1.80 (1.23-2.64)	.003	1.26 (0.81-1.95)	.31	1.16 (0.71-1.90)	.54	1.01 (0.59-1.73)	.97
32 wk	5325	2.29 (1.63-3.22)	<.001	1.82 (1.16-2.87)	.009	1.90 (1.15-3.14)	.01	1.41 (0.81-2.45)	.22
Postnatal									
8 wk	5189	2.38 (1.62-3.46)	<.001	1.84 (1.21-2.80)	.005	1.83 (1.15-2.93)	.01	1.75 (1.04-2.94)	.03
8 mo	5132	2.25 (1.50-3.39)	<.001	1.76 (1.13-2.75)	.01	1.80 (1.10-2.95)	.02	1.65 (0.95-2.85)	.08
**Offspring aged 15 y**									
Antenatal									
18 wk	3427	1.59 (0.98-2.58)	.06	1.31 (0.75-2.27)	.34	1.28 (0.70-2.32)	.42	1.31 (0.70-2.42)	.39
32 wk	3568	2.11 (1.38-3.22)	.001	2.04 (1.78-3.53)	.01	2.17 (1.21-3.86)	.009	1.88 (1.02-3.48)	.04
Postnatal									
8 wk	3472	1.79 (1.08-2.98)	.02	1.59 (0.93-2.74)	.09	1.82 (1.02-3.24)	.04	1.59 (0.87-2.91)	.13
8 mo	3417	2.26 (1.37-3.72)	.001	2.02 (1.18-3.46)	.01	1.92 (1.07-3.44	.03	1.92 (1.05-3.51)	.04

Abbreviations: ODD, oppositional defiant disorder; OR, odds ratio.

^a^ Unadjusted.

^b^ Adjusted for maternal age, income, educational status, ethnicity, parity, prepregnancy body mass index, pregnancy diabetes, urinary tract infection during pregnancy, preeclampsia, alcohol consumption during pregnancy, smoking during pregnancy, and anxiety and offspring sex and gestational age at delivery.

^c^ Adjusted for covariates in model 2 and comorbid conduct disorder in each age group.

^d^ Adjusted for covariates in model 2 and comorbid conduct and attention-deficit/hyperactivity disorder in each age group.

## Discussion

In this population-based longitudinal birth cohort study, we investigated the risk of ODD in children and adolescents of mothers with perinatal depressive symptoms. After accounting for a wide range of known confounders and comorbid disorders, we found associations between maternal perinatal depressive symptoms and an increased risk of ODD in offspring. Specifically, persistent depressive symptoms during pregnancy and in the first year of the postpartum period were associated with a 4-fold increased risk of ODD in offspring over time when compared with offspring who were not exposed. Analyses at each time point also showed consistent results and suggested that these associations track from early childhood to middle childhood and adolescence. Our sensitivity and attrition analyses suggest the robustness of the findings observed in the main analysis.

To our knowledge, no previous studies have examined the association between maternal depressive symptoms during pregnancy and the postpartum period and the risk of ODD in children and adolescents; however, several studies have explored the association between maternal perinatal depression and offspring behavioral and emotional problems. Consistent with our results, these studies have found a positive association between maternal perinatal depressive symptoms and adverse emotional and behavioral outcomes in offspring.^13,14,15,33^ For example, in a previous study based on the ALSPAC data,^15^ a 33% increased risk of attention problems was found in children whose mothers had depressive symptoms during pregnancy (OR, 1.33; 95% CI, 1.19-1.48). A 2018 population-based pregnancy cohort study by Kingston et al^14^ also found that children whose mothers had persistent high levels of perinatal depressive symptoms during pregnancy and the first 12 months post partum were more likely to report externalizing problems when compared with children who were not exposed, which is in line with the findings of our study. Our findings, together with existing evidence, suggest that antenatal and postnatal depressive symptoms are associated with an increased risk of child behavior problems and that persistent maternal perinatal depressive symptoms are associated with the highest risk.

The possible mechanisms underlying the observed association may include genetic, epigenetic, environmental, and psychological factors.^8,9,10,11^ A number of genome-wide association studies^9,34,35,36^ have reported genetic correlations between depressive disorders and other common psychiatric disorders. Polygenic risk scores for neurodevelopmental disorders are also associated with a number of maternal perinatal conditions, including depression.^37^ For example, conduct disorder and ADHD, the most common comorbid conditions with ODD, appear to be genetically correlated with depressive symptoms, suggesting shared genetic vulnerability.^38,39^ Antenatal depression might also exert an effect through intrauterine mechanisms. One proposed mechanism is through altering the maternal hypothalamic-pituitary-adrenal axis activity,^40^ which might have effects on placental function, fetal development, epigenetics, and immune function, all of which have been implicated in the etiology of emotional and behavioral disorders in offspring.^41,42^ The relation between maternal postnatal depression and offspring externalizing behaviors has been explained, in part, by the effect of depression on maternal sensitivity toward the child, the security of the attachment, and parenting,^42,43^ which might, in turn, increase the risk of emotional and behavioral problems such as ODD in offspring.^13,42^ Maternal antenatal depression might also interfere with mother-infant attachment in the early postnatal period.^43^ An association between antenatal and postnatal depression and increased risk of insecure mother-infant attachment has been reported widely.^44,45^

### Limitations

This study has some limitations. First, attrition, as with all cohort studies, can introduce selection bias. In comparison with those retained in the analyses, mothers of children who were lost to follow-up or missing data were younger at childbirth and less educated and were more likely to be overweight/obese, have urinary tract infections during pregnancy, smoke tobacco, and have higher levels of antenatal anxiety symptoms (eTable 3 in the Supplement). Because these factors are also associated with adverse mental health and behavioral outcomes in offspring,^25,26,27,28^ it has been argued that this type of loss to follow-up is more likely to weaken the point estimates of any observed association between exposures and outcome. Previous work in ALSPAC^46^ has also suggested that selective dropout does not bias the prediction of risk of behavioral disorders. Consistent with this, a recent longitudinal study showed that loss to follow-up rarely affects estimates of associations.^47^ In addition, when we used multiple imputations to address attrition bias, estimates from multiple imputations and complete case analyses were broadly comparable (eTable 8 in the Supplement), suggesting that attrition due to missing data was unlikely to have biased our results. Second, we relied on measures of depressive symptoms rather than a diagnostic instrument. This may lead to random measurement error. However, EPDS is a valid and reliable tool for assessing maternal depressive symptoms and has been widely used in research and clinical practice.^21^ In addition, any random measurement error would tend to reduce the size of the association rather than lead to spurious associations. Finally, although we adjusted for potential confounders, including comorbid conduct disorder and ADHD, our findings may still be influenced by unmeasured genetic, epigenetic, and environmental confounders. For example, maternal mental health and stressful life events before pregnancy are major determinants of perinatal depression and are associated with offspring psychiatric and neurodevelopmental disorders.^11,48,49,50^ These and other unmeasured confounders could bias the association between maternal perinatal depressive symptoms and offspring ODD away from the null.

## Conclusions

In this population-based longitudinal cohort study, we found an association between mothers with depressive symptoms during the perinatal period and offspring who have with ODD during childhood and adolescence, although residual and unmeasured confounding by environmental and genetic factors warrants further study. These findings support demands for identifying and managing maternal depression at the time of pregnancy, especially for women with postnatal and/or persistent depressive symptoms.

## Notes

Supplement. **eMethods.** Cohort Description **eFigure.** Study Participants Included in the Analysis **eTable 1.** Two-by-Two Table: Summary of Exposure and Outcome Data Included in the Final Analysis by Age Group **eTable 2.** Missingness Distributions of Outcomes, Exposures, and Major Covariates **eTable 3.** Study Participants With Complete Data on Offspring ODD Compared With Those With Missed or Loss to Follow-up **eTable 4.** Maternal Antenatal and Postnatal Depressive Symptoms and Risk of ODD in Offspring Over Time After Further Adjustment to Comorbid Depression Disorders in Offspring **eTable 5.** Maternal Antenatal and Postnatal Depressive Symptoms and Risk of ODD in Offspring Over Time Using Continuous EPDS Scores (GEE Models) **eTable 6.** Association Between Persistent Depressive Symptoms and Risk of ODD in Offspring Over Time **eTable 7.** Maternal Antenatal and Postnatal Depressive Symptoms and Risk of ODD in Offspring at Each Time Point Using Continuous EPDS Scores (Logistic Regression Analysis) **eTable 8.** Maternal Antenatal and Postnatal Depressive Symptoms and Risk of ODD in Offspring at Each Time (Using Imputed Datasets) **eTable 9.** Maternal Antenatal and Postnatal Depressive Symptoms and Risk of ODD in Offspring (Using Imputed Datasets)
eReferences
