# Supplementary material for: Association of Maternal Depressive Symptoms During the Perinatal Period With Oppositional Defiant Disorder in Children and Adolescents
Source: JAMA Netw Open. 2021 Sep 30;4(9):e2125854. doi: 10.1001/jamanetworkopen.2021.25854 (PMC8485171; doi:10.1001/jamanetworkopen.2021.25854)
Supplement: Supplement. — eMethods. Cohort Description eFigure. Study Participants Included in the Analysis eTable 1. Two-by-Two Table: Summary of Exposure and Outcome Data Included in the Final Analysis by Age Group eTable 2. Missingness Distributions of Outcomes, Exposures, and Major Covariates eTable 3. Study Participants With Complete Data on Offspring ODD Compared With Those With Missed or Loss to Follow-up eTable 4. Maternal Antenatal and Postnatal Depressive Symptoms and Risk of ODD in Offspring Over Time After Further Adjustment to Comorbid Depression Disorders in Offspring eTable 5. Maternal Antenatal and Postnatal Depressive Symptoms and Risk of ODD in Offspring Over Time Using Continuous EPDS Scores (GEE Models) eTable 6. Association Between Persistent Depressive Symptoms and Risk of ODD in Offspring Over Time eTable 7. Maternal Antenatal and Postnatal Depressive Symptoms and Risk of ODD in Offspring at Each Time Point Using Continuous EPDS Scores (Logistic Regression Analysis) eTable 8. Maternal Antenatal and Postnatal Depressive Symptoms and Risk of ODD in Offspring at Each Time (Using Imputed Datasets) eTable 9. Maternal Antenatal and Postnatal Depressive Symptoms and Risk of ODD in Offspring (Using Imputed Datasets) eReferences [file jamanetwopen-e2125854-s001.pdf]

Supplementary Online Content

Dachew BA, Scott JG, Heron JE, Ayano G, Alati R. Association of maternal depressive symptoms during the perinatal period with oppositional defiant disorder in children and adolescents. *JAMA Netw Open*. 2021;4(9):e2125854.
doi:10.1001/jamanetworkopen.2021.25854

eMethods. Cohort Description
eFigure. Study Participants Included in the Analysis
eTable 1. Two-by-Two Table: Summary of Exposure and Outcome Data Included in the Final Analysis by Age Group
eTable 2. Missingness Distributions of Outcomes, Exposures, and Major Covariates
eTable 3. Study Participants With Complete Data on Offspring ODD Compared With Those With Missed or Loss to Follow-up
eTable 4. Maternal Antenatal and Postnatal Depressive Symptoms and Risk of ODD in Offspring Over Time After Further Adjustment to Comorbid Depression Disorders in Offspring
eTable 5. Maternal Antenatal and Postnatal Depressive Symptoms and Risk of ODD in Offspring Over Time Using Continuous EPDS Scores (GEE Models)
eTable 6. Association Between Persistent Depressive Symptoms and Risk of ODD in Offspring Over Time
eTable 7. Maternal Antenatal and Postnatal Depressive Symptoms and Risk of ODD in Offspring at Each Time Point Using Continuous EPDS Scores (Logistic Regression Analysis)
eTable 8. Maternal Antenatal and Postnatal Depressive Symptoms and Risk of ODD in Offspring at Each Time (Using Imputed Datasets)
eTable 9. Maternal Antenatal and Postnatal Depressive Symptoms and Risk of ODD in Offspring (Using Imputed Datasets)
eReferences

This supplementary material has been provided by the authors to give readers additional information about their work.

eMethods. Cohort Description

All pregnant women residents in Avon, UK, with expected delivery dates between 01 April 1991 to 31 December 1992, were invited to participate in the study. The initial number of pregnancies enrolled is 14,541 (for these at least one questionnaire has been returned, or a "Children in Focus" clinic had been attended by 19/07/99). Of these initial pregnancies, there were a total of 14,676 foetuses, resulting in 14,062 live births and 13,988 children who were alive at 1 year of age.[1-3] A further 456 children from 452 pregnancies and 257 children from 254 pregnancies were recruited postnatally at age seven and from age eight, respectively, resulting in 15,247 enrolled pregnancies. When compared with the 1991 National Census Data, the ALSPAC sample was found to be similar to the whole UK population.[1] Further details regarding recruitment, study design, and generalizability have been previously reported [1,2], and the study website contains information of all the data that is available through a fully searchable data dictionary and variable search tool.[4]

We had complete data on maternal antenatal depression assessed at 18 weeks of gestation for 11657 mothers and at 32 weeks of gestation for 11719 mothers. Data on postnatal depressive symptoms at eight weeks and eight months were available for 11429 and 10923 mothers, respectively. Data on offspring ODD at age 7, 10, 13 and 15 years were available for 7988, 7588, 6886, and 4630 children, respectively. The final analyses were conducted in children with complete data on exposure and outcome (at each time point) and potential confounders (eFigure 1 and eTable 1).

© 2021 Dachew BA et al. *JAMA Network Open*.

eFigure. Study Participants Included in the Analysis

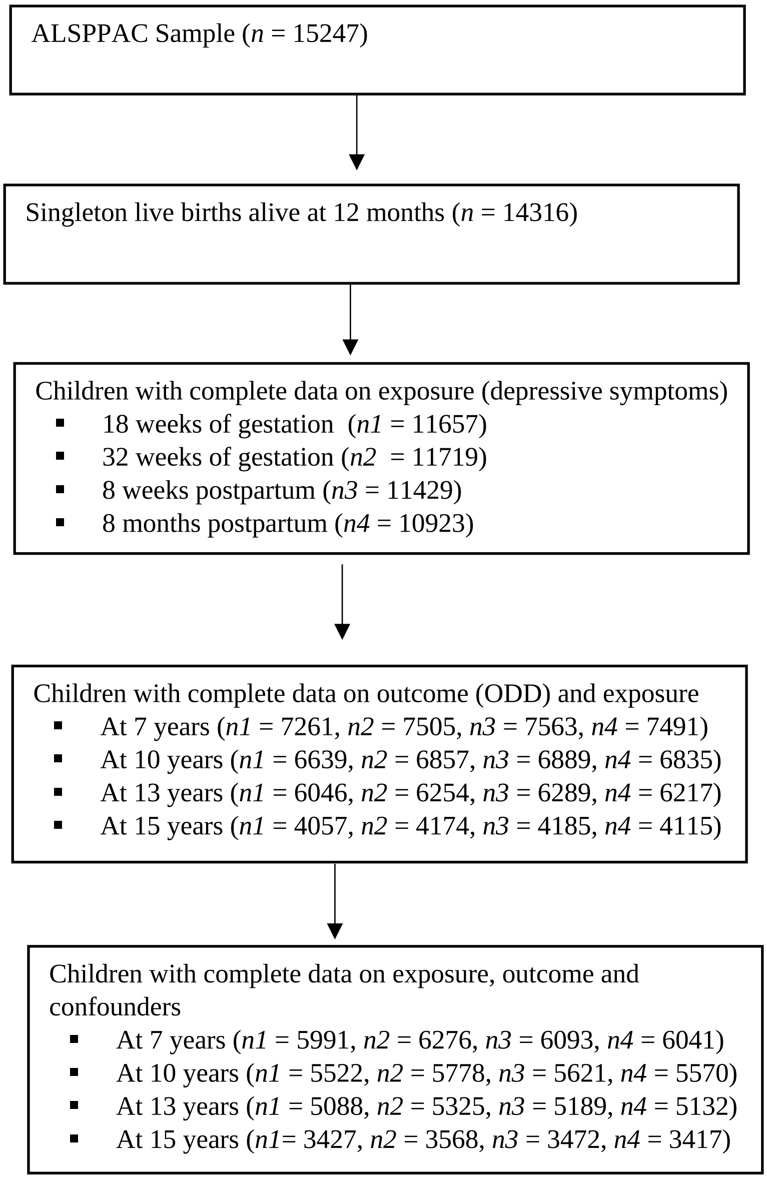

© 2021 Dachew BA et al. *JAMA Network Open*.

eTable 1. Two-by-Two Table: Summary of Exposure and Outcome Data Included in the Final Analysis by Age Group

Offspring age	Depressive symptoms		Offspring ODD	
			Yes	No
			n (%)	*n* (%)
7 years	18 weeks of gestation (*n* = 5991)	Yes	49 (5.9)	786 (94.1)
		No	148 (2.9)	5008 (97.1)
		Total	197 (3.3)	5794 (96.7)
	32 weeks of gestation (*n* = 6276)	Yes	65 (6.3)	973 (93.7)
		No	143 (2.7)	5095 (97.3)
		Total	208 (3.3)	6068 (96.7)
	8 weeks postnatally (*n* = 6093)	Yes	63 (9.1)	632 (90.9)
		No	142 (2.6)	5256 (97.4)
		Total	205 (3.4)	5888 (96.4)
	8 months postnatally (*n* = 6041)	Yes	49 (8.4)	533 (91.6)
		No	148 (2.7)	5311 (97.3)
		Total	197 (3.3)	5884 (96.7)
10 years	18 weeks of gestation (*n* = 5522)	Yes	45 (6.0)	702 (94.0)
		No	132 (2.8)	4643 (9.2)
		Total	177 (3.2)	5345 (96.8)
	32 weeks of gestation (*n* = 5778)	Yes	60 (6.6)	849 (93.4)
		No	130 (2.7)	4739 (97.3)
		Total	190 (3.3)	5588 (96.7)
	8 weeks postnatally (*n* = 5621)	Yes	45 (7.2)	580 (92.8)
		No	140 (2.8)	4856 (97.2)
		Total	185 (3.3)	5436 (96.7)
	8 months postnatally (*n* = 5570)	Yes	44 (8.5)	477 (91.2)
		No	140 (2.8)	4909 (97.2)
		Total	184 (3.3)	5386 (96.7)
13 years	18 weeks of gestation (*n* = 5008)	Yes	35 (5.0)	668 (95.0)
		No	124 (2.8)	4261 (97.2)
		Total	159 (3.1)	4929 (96.9)
	32 weeks of gestation (*n* = 5325)	Yes	49 (5.9)	780 (94.1)
		No	120 (2.7)	4376 (97.3)
		Total	169 (3.2)	5156 (96.8)
	8 weeks postnatally (*n* = 5189)	Yes	36 (6.4)	528 (93.6)
		No	129 (2.8)	4496 (97.2)
		Total	165 (3.2)	5024 (96.8)
	8 months postnatally (*n* = 5132)	Yes	30 (6.3)	449 (93.7)
		No	134 (2.9)	4519 (97.1)
		Total	164 (3.2)	4968 (96.8)
15 years	18 weeks of gestation (*n* = 3427)	Yes	21 (4.6)	438 (95.4)
		No	87 (2.9)	2881 (97.1)
		Total	108 (3.2)	3319 (96.8)
	32 weeks of gestation (*n* = 3568)	Yes	31 (5.7)	514 (94.3)
		No	84 (2.8)	2939 (97.2)
		Total	115 (3.2)	3453 (96.8)

© 2021 Dachew BA et al. *JAMA Network Open*.

	8 weeks postnatally (*n* = 3472)	Yes	19 (5.2)	347 (94.8)
		No	92 (3.0)	3014 (97.0)
		Total	111 (3.2)	3361 (96.8)
	8 months postnatally (*n* = 3417)	Yes	20 (6.4)	293 (93.6)
		No	91 (2.9)	3013 (97.1)
		Total	111 (3.2)	3306 (96.8)

eTable 2. Missingness Distributions of Outcomes, Exposures, and Major Covariates

Characteristics	Total	Overall missing *n* (%)	Among participants with complete outcome data *n* (%)			
			7 years (*n* = 7998)	10 years (*n* = 7588)	13 years (*n* = 6886)	15 years (*n* = 4630)
Maternal age at delivery	14316	703 (4.9)	31 (0.39)	337 (4.4)	296 (4.3)	218 (4.7)
Maternal education	14316	2965 (20.7)	548 (6.90	780 (10.3)	664 (9.6)	467 (10.1)
Parity	14316	1719 (12.0)	271 (3.4)	533 (7.0)	460 (6.7)	234 (7.5)
Pre-pregnancy BMI	14316	3064 (21.4)	732 (9.2)	980 (12.9)	833 (12.1)	599 (12.9)
Hypertensive disorders during pregnancy	14316	919 (6.4)	66 (0.83)	369 (4.9)	327 (4.8)	238 (5.1)
Pregnancy diabetes status	14316	2272 (15.9)	284 (3.6)	561 (7.4)	488 (7.1)	362 (7.8)
Urinary tract infection during pregnancy	14316	2483 (17.3)	433 (5.4)	683 (9.0)	594 (8.6)	424 (9.2)
Alcohol drinking in pregnancy	14316	1641 (11.5)	208 (2.6)	483 (6.4)	417 (6.1)	308 (6.7)
Smoking during pregnancy	14316	1494 (10.4)	168 (2.1)	447 (5.9)	390 (5.7)	294 (6.4)
Maternal antenatal anxiety symptoms	14316	2901 (20.3)	654 (8.2)	878 (11.6)	768 (11.2)	536 (11.6)
Maternal depressive symptoms						
18 weeks of gestation	14316	2659 (18.6)	737 (9.2)	949 (12.5)	840 (12.2)	573 (12.4)
32 weeks of gestation	14316	2597 (18.1)	493 (6.2)	731 (9.6)	632 (9.2)	456 (9.9)
8 weeks postpartum	14316	2887 (20.2)	435 (5.4)	699 (9.2)	597 (8.7)	445 (9.6)
8 months postpartum	14316	3393 (23.7)	507 (6.3)	753 (9.9)	669 (9.7)	515 (11.1)
Offspring sex	14316	5 (0.03)	4 (0.05)	2 (0.03)	2 (0.03)	2 (0.04)
Gestational age at delivery	14316	703 (4,9)	31 (0.39)	337 (4.4)	296 (4.3)	218 (4.7)
Offspring ODD						
At 7 years	14316	6318 (44.1)	-	1121 (14.8)	1014 (14.7)	734 (15.9)
At 10 years	14316	6728 (47.0)	1531 (19.1)	-	772 (11.2)	579 (12.5)
At 13 years	14316	7430 (51.9)	2126 (26.6)	1474 (19.4)	-	586 (12.7)
At 15 years	14316	9686 (67.7)	4102 (51.3)	3537 (46.6)	2842 (41.3)	-

© 2021 Dachew BA et al. *JAMA Network Open*.

eTable 3. Study Participants With Complete Data on Offspring ODD Compared With Those With Missed or Loss to Follow-up

Characteristics	7 years		P	10 years		P	13 years		P	15 years		p
	Complete data on ODD (n = 7998)	Missed/lost follow-up *(n = 6318)*		Complete data on ODD (n = 7588)	Missed/lost follow-up (n = 6728)		Complete data on ODD (*(n = 6886)*	Missed/lost follow-up (*n*=7430)		Complete data on ODD (n = 4630)	Missed/lost follow-up (*n*=9686)	
Maternal age at delivery (mean, SD)	28.6 (4.6)	26.4 (5.1)	<0.001	28.7 (4.6)	26.6 (5.1)	<0.001	28.8 (4.6)	26.7 (5.0)	<0.001	28.9 (4.5)	27.1 (5.0)	<0.001
Maternal education (CSE, %)	11.0	22.3	<0.001	10.5	21.5	<0.001	9.8	21.2	<0.001	8.0	18.9	<0.001
Parity (nullipara, %)	46.3	42.8	<0.001	46.5	42.9	<0.001	47.4	42.2	<0.001	49.3	42.6	<0.001
Pre-pregnancy BMI (≥25Kg/m2, %)	19.7	22.6	<0.001	19.6	22.3	<0.001	19.3	22.3	<0.001	19.1	21.6	<0.001
HDP during pregnancy (Yes, %)	15.9	16.6	0.23	15.8	16.6	0.20	16.2	16.1	0.91	16.3	16.1	0.81
Pregnancy diabetes status (Yes, %)	4.1	4.1	0.95	3.9	4.3	0.30	3.9	4.3	0.34	4.0	4.1	0.77
UTI during pregnancy (Yes, %)	5.4	8.1	<0.001	5.3	7.9	<0.001	5.2	7.7	<0.001	5.6	6.8	0.009

© 2021 Dachew BA et al. *JAMA Network Open*.

Alcohol drinking in pregnancy (Yes, %)	15.4	16.4	0.05	15.3	16.6	0.04	15.2	16.5	0.04	15.1	16.3	0.08
Smoking during pregnancy (Yes, %)	19.3	33.8	<0.001	18.2	33.4	<0.001	17.5	32.6	<0.001	15.0	30.0	<0.001
Maternal antenatal anxiety symptoms (Yes, %)	20.1	26.9	<0.001	19.7	27.2	<0.001	19.7	26.4	<0.001	19.1	24.9	<0.001
Maternal depressive symptoms												
18 weeks of gestation (Yes, %)	14.8	22.7	<0.001	14.3	22.3	<0.001	14.6	21.2	<0.001	13.8	19.9	<0.001
32 weeks of gestation (Yes, %)	17.1	23.8	<0.001	16.5	23.7	<0.001	16.3	23.2	<0.001	15.8	21.6	<0.001
8 weeks postpartum (Yes, %)	11.8	15.5	<0.001	11.5	15.3	<0.001	11.3	15.1	<0.001	10.8	14.3	<0.001
8 months postpartum (Yes, %)	10.2	13.3	<0.001	10.0	13.1	<0.001	10.0	12.7	<0.001	9.6	12.1	<0.001
Offspring sex (Male, %)	51.3	51.2	0.86	50.4	52.2	0.03	50.2	52.2	0.02	47.9	52.9	<0.001
Gestational age at delivery (<37 weeks, %)	4.3	5.4	<0.02	4.3	5.3	0.01	4.1	5.3	0.001	4.3	5.0	0.09

Certificate of Secondary Education (CSE); Hypertensive disorders of pregnancy (HDP); Urinary tract infection (UTI). P-values correspond to Pearson's chi-square test for categorical variables and t-test for numerical variables.

© 2021 Dachew BA et al. *JAMA Network Open*.

eTable 4. Maternal Antenatal and Postnatal Depressive Symptoms and Risk of ODD in Offspring Over Time After Further Adjustment to Comorbid Depression Disorders in Offspring

Maternal depressive symptoms	Unadjusted (Model 1) OR (95% CI)	*P*	Final model (Model 4)# OR (95% CI)	*P*	Model 5 OR (95% CI)	*P*
18 Weeks of gestation	2.09 (1.66-2.64)	<0.001	1.25 (0.94-1.65)	0.13	1.18 (0.88 – 1.57)	0.27
32 weeks of gestation	2.40 (1.94-2.95)	<0.001	1.43 (1.07-1.92)	0.02	1.41 (1.04 – 1.90)	0.02
8 weeks postnatally	2.94 (2.35-3.68)	<0.001	1.87 (1.42-2.45)	<0.001	1.83 (1.39 – 2.41)	<0.001
8 months postnatally	2.78 (2.19-3.54)	<0.001	1.93 (1.45-2.56)	<0.001	1.93 (1.45 – 2.57)	<0.001

Model 4 adjusted for maternal age, income, educational status, ethnicity, parity, pre-pregnancy BMI, pregnancy diabetes, urinary tract infection (UTI) during pregnancy, pre-eclampsia, alcohol drinking during pregnancy, smoking during pregnancy, maternal anxiety, offspring sex and gestational ages at delivery, and comorbid CD, ADHD over time.
Model 5: Additionally adjusted for comorbid depression over time.

© 2021 Dachew BA et al. *JAMA Network Open*.

eTable 5. Maternal Antenatal and Postnatal Depressive Symptoms and Risk of ODD in Offspring Over Time Using Continuous EPDS Scores (GEE Models)

Depressive symptoms	Number of observations#	Model 1 OR (95% CI)	*P*	Model 2 OR (95% CI)	*P*	Model 3 OR (95% CI)	*P*	Model 4	*P*
18 weeks of gestation	20028	1.09 (1.06-1.10)	<0.001	1.05 (1.02-1.07)	<0.001	1.04 (1.01-1.06)	0.003	1.00 (1.01-1.06)	0.02
32 weeks of gestation	20947	1.09 (1.07-1.11)	<0.001	1.07 (1.05-1.10)	<0.001	1.07 (1.04-1.09)	<0.001	1.05 (1.03-1.08)	<0.001
8 weeks postnatally	20372	1.10 (1.08-1.12)	<0.001	1.08 (1.05-1.10)	<0.001	1.07 (1.05-1.09)	<0.001	1.06 (1.04-1.08)	<0.001
8 months postnatally	20160	1.09 (1.07-1.11)	<0.001	1.07 (1.05-1.10)	<0.001	1.06 (1.04-1.09)	<0.001	1.06 (1.04-1.08)	<0.001

Model 1 was unadjusted; Model 2 adjusted for maternal age, income, educational status, ethnicity, parity, pre-pregnancy BMI, pregnancy diabetes, urinary tract infection (UTI) during pregnancy, pre-eclampsia, alcohol drinking during pregnancy, smoking during pregnancy, maternal anxiety, offspring sex and gestational ages at delivery; Model 3 further adjusted for comorbid CD over time and Model 4 additionally adjusted for comorbid ADHD over time.

#The number of observations over time refers to the sum of the samples included at each age group (over 4-time points). For example, for antenatal depressives symptoms measured at 18 weeks of gestation, a total of 5991 (at 7 years), 5222 (at 10 years), 5008 (at 13 years) and 3427 (at 15 years) participants were included (see eFigure for details).

© 2021 Dachew BA et al. *JAMA Network Open*.

eTable 6. Association Between Persistent Depressive Symptoms and Risk of ODD in Offspring Over Time

Persistent depressive symptoms	Model 1 OR (95% CI)	*P*	Model 2 OR (95% CI)	*P*	Model 3 OR (95% CI)	*P*	Model 4	*P*
Yes (*n* = 221)	5.60 (3.65 – 8.59)	< 0.001	3.59 (1.98 – 6.52)	< 0.001	3.21 (1.70 -6.07)	< 0.001	2.85 (1.51 – 5.39)	0.001
No (*n* = 6277)	1 (Reference)		1 (Reference)		1 (Reference)		1 (Reference)	

Model 1 was unadjusted; Model 2 adjusted for maternal age, income, educational status, ethnicity, parity, pre-pregnancy BMI, pregnancy diabetes, urinary tract infection (UTI) during pregnancy, pre-eclampsia, alcohol drinking during pregnancy, smoking during pregnancy, maternal anxiety, offspring sex and gestational ages at delivery; Model 3 further adjusted for comorbid CD over time, and Model 4 additionally adjusted for comorbid ADHD over time.

© 2021 Dachew BA et al. *JAMA Network Open*.

eTable 7. Maternal Antenatal and Postnatal Depressive Symptoms and Risk of ODD in Offspring at Each Time Point Using Continuous EPDS Scores (Logistic Regression Analysis)

Offspring age	Depressive symptoms	Model 1<br>OR (95% CI)	*P*	Model 2<br>OR (95% CI)	*P*	Model 3<br>OR (95% CI)	*P*	Model 4<br>OR (95% CI)	*P*
7 years	Antenatal								
	18 weeks [*n* = 5991]	1.07 (1.04-1.10)	<0.001	1.03 (0.99-1.06)	0.09	1.02 (0.98-1.05)	0.39	1.00 (0.96-1.04)	0.97
	32 weeks [*n* = 6276]	1.08 (1.06-1.11)	<0.001	1.05 (1.01-1.09)	0.007	1.05 (1.01-1.09)	0.01	1.03 (0.99-1.07)	0.15
	Postnatal								
	8 weeks [*n* = 6093]	1.11 (1.09-1.14)	<0.001	1.09 (1.06-1.12)	<0.001	1.08 (1.05-1.12)	<0.001	1.07 (1.04-1.12)	<0.001
	8 months [*n* = 6041]	1.11 (1.08-1.14)	<0.001	1.09 (1.06-1.12)	<0.001	1.08 (1.05-1.12)	<0.001	1.08 (1.04-1.12)	<0.001
10 years	Antenatal								
	18 weeks [*n* = 5522]	1.10 (1.08-1.14)	<0.001	1.08 (1.05-1.12)	<0.001	1.07 (1.03-1.11)	0.001	1.06 (1.02-1.11)	0.004
	32 weeks [*n* = 5778]	1.10 (1.07-1.13)	<0.001	1.09 (1.05-1.13)	<0.001	1.08 (1.04-1.12)	<0.001	1.06 (1.02-1.11)	0.004
	Postnatal								
	8 weeks [*n* = 5621]	1.09 (1.07-1.12)	<0.001	1.07 (1.04-1.11)	<0.001	1.07 (1.03-1.11)	<0.001	1.06 (1.03-1.10)	0.001
	8 months [*n* = 5570]	1.11 (1.08-1.14)	<0.001	1.09 (1.06-1.12)	<0.001	1.08 (1.05-1.12)	<0.001	1.08 (1.04-1.12)	<0.001
13 years	Antenatal								
	18 weeks [*n* = 5088]	1.07 (1.04-1.11)	<0.001	1.05 (1.01-1.08)	0.02	1.04 (0.99-1.08)	0.08	1.04 (10.99-1.08)	0.11
	32 weeks [*n* = 5325]	1.11 (1.07-1.13)	<0.001	1.09 (1.05-1.14)	<0.001	1.09 (1.05-1.14)	<0.001	1.08 (1.03-1.12)	0.002

© 2021 Dachew BA et al. *JAMA Network Open*.

	Postnatal								
	8 weeks [*n* = 5189]	1.08 (1.05-1.11)	<0.001	1.06 (1.02-1.09)	0.001	1.05 (1.01-1.09)	0.01	1.05 (1.01-1.09)	0.03
	8 months [*n* = 5132]	1.08 (1.04-1.11)	<0.001	1.06 (1.02-1.09)	0.001	1.05 (1.01-1.09)	0.007	1.05 (1.01-1.09)	0.02
15 years	Antenatal								
	18 weeks [*n* = 3427]	1.05 (1.01-1.10)	0.009	1.04 (0.99-1.09)	0.10	1.03 (0.98-1.08)	0.29	1.02 (0.97-1.08)	0.42
	32 weeks [*n* = 3568]	1.08 (1.04-1.12)	<0.001	1.09 (1.04-1.14)	<0.001	1.08 (1.03-1.14)	0.002	1.07 (1.02-1.13)	0.007
	Postnatal								
	8 weeks [*n* = 3472]	1.06 (1.02-1.10)	0.002	1.06 (1.01-1.10)	0.01	1.06 (1.01-1.11)	0.01	1.05 (1.00-1.10)	0.05
	8 months [*n* = 3417]	1.06 (1.03-1.11)	0.001	1.06 (1.01-1.10)	0.01	1.05 (1.01-1.10)	0.03	1.05 (1.00-1.10)	0.04

Model 1 was unadjusted; Model 2 adjusted for maternal age, income, educational status, ethnicity, parity, pre-pregnancy BMI, pregnancy diabetes, urinary tract infection (UTI) during pregnancy, pre-eclampsia, alcohol drinking during pregnancy, smoking during pregnancy, maternal anxiety, offspring sex and gestational ages at delivery; Model 3 further adjusted for comorbid CD in each age group, and Mode1 4 additionally adjusted for comorbid ADHD in each age group.

© 2021 Dachew BA et al. *JAMA Network Open*.

eTable 8. Maternal Antenatal and Postnatal Depressive Symptoms and Risk of ODD in Offspring at Each Time (Using Imputed Datasets)

Offspring age	Depressive symptoms	Model 1 OR (95% CI)	*P*	Model 2 OR (95% CI)	*P*	Model 3 OR (95% CI)	*P*	Model 4 OR (95% CI)	*P*
7 years	Antenatal								
	18 weeks [*n* = 7261]	2.09 (1.56-2.81)	<0.001	1.30 (0.93-1.82)	0.12	1.16 (0.81-1.67)	0.42	1.11 (0.74-1.66)	0.63
	32 weeks [*n* = 7505]	2.38 (1.81-3.12)	<0.001	1.46 (1.03-2.07)	0.04	1.43 (0.99-2.07)	0.06	1.17 (0.76-1.78)	0.48
	Postnatal								
	8 weeks [*n* = 7563]	3.38 (2.56-4.45)	<0.001	2.54 (1.86-3.45)	<0.001	2.42 (1.74-3.36)	<0.001	2.30 (1.59-3.35)	<0.001
	8 months [*n* = 7491]	3.27 (2.43-4.40)	<0.001	2.47 (1.79-3.42)	<0.001	2.31 (1.64-3.28)	<0.001	2.33 (1.57-3.44)	<0.001
10 years	Antenatal								
	18 weeks [*n* = 6639]	2.09 (1.52-2.87)	<0.001	1.45 (1.01-2.07)	0.04	1.28 (0.86-1.89)	0.23	1.21 (0.78-1.89)	0.32
	32 weeks [*n* = 6857]	2.67 (2.01-3.54)	<0.001	2.11 (1.46-3.04)	<0.001	1.95 (1.31-2.90)	0.001	1.84 (1.19-2.85)	0.01
	Postnatal								
	8 weeks [*n* = 6889]	2.53 (1.85-3.46)	<0.001	2.01 (1.42-2.83)	<0.001	1.96 (1.35-2.85)	<0.001	1.69 (1.11-2.59)	0.02
	8 months [*n* = 6835]	3.17 (2.32-4.32)	<0.001	2.59 (1.84-3.64)	<0.001	2.33 (1.60-3.39)	<0.001	2.10 (1.38-3.20)	0.001
13 years	Antenatal								
	18 weeks [*n* = 6046]	2.16 (1.55-3.01)	<0.001	1.62 (1.11-2.34)	0.01	1.55 (1.03-2.43)	0.04	1.39 (0.88-2.20)	0.16

© 2021 Dachew BA et al. *JAMA Network Open*.

	32 weeks [*n* = 6254]	2.38 (1.76-3.23)	<0.001	1.88 (1.27-2.79)	0.002	1.84 (1.19-2.84)	0.006	1.53 (0.94-2.50)	0.09
	Postnatal								
	8 weeks [*n* = 6289]	2.51 (1.80-3.49)	<0.001	1.97 (1.37-2.85)	<0.001	1.81 (1.21-2.72)	0.004	1.79 (1.13-2.84)	0.01
	8 months [*n* = 6217]	2.46 (1.73-3.49)	<0.001	1.86 (1.27-2.73)	0.001	1.86 (1.22-2.83)	0.004	1.85 (1.16-2.96)	0.01
15 years	Antenatal								
	18 weeks [*n* = 4057]	1.75 (1.16-2.65)	0.008	1.44 (0.90-2.29)	0.13	1.47 (0.89-2.43)	0.14	1.44 (0.86-2.43)	0.17
	32 weeks [*n* = 4174]	1.96 (1.35-2.85)	<0.001	1.70 (1.05-2.73)	0.03	1.73 (1.03-2.90)	0.04	1.65 (0.96-2.84)	0.07
	Postnatal								
	8 weeks [*n* = 4185]	1.84 (1.20-2.83)	0.005	1.59 (1.00-2.53)	0.05	1.67 (1.01-2.75)	0.05	1.52 (0.90-2.58)	0.12
	8 months [*n* = 4115]	2.74 (1.83-4.12)	<0.001	2.45 (1.57-3.82)	<0.001	2.44 (1.50-3.97)	<0.001	2.56 (1.55-4.23)	<0.001

Model 1 was unadjusted; Model 2 adjusted for maternal age, income, educational status, ethnicity, parity, pre-pregnancy BMI, pregnancy diabetes, urinary tract infection (UTI) during pregnancy, pre-eclampsia, alcohol drinking during pregnancy, smoking during pregnancy, maternal anxiety, offspring sex and gestational ages at delivery; Model 3 further adjusted for comorbid CD in each age group, and Model 4 additionally adjusted for comorbid ADHD in each age group.

complete exposure and outcome data and imputed missing covariates

© 2021 Dachew BA et al. *JAMA Network Open*.

eTable 9. Maternal Antenatal and Postnatal Depressive Symptoms and Risk of ODD in Offspring (Using Imputed Datasets)

Offspring age	Depressive symptoms	Model 1 OR (95% CI)	*P*	Model 2 OR (95% CI)	*P*	Model 3 OR (95% CI)	*P*	Model 4 OR (95% CI)	*P*
7 years	Antenatal								
	18 weeks [*n* = 11657]	2.05 (1.58-2.66)	<0.001	1.19 (0.88-1.60)	0.25	1.11 (0.80-1.54)	0.51	1.06 (0.76-1.52)	0.70
	32 weeks [*n* = 11719]	2.45 (1.92-3.12)	<0.001	1.27 (0.94-1.72)	0.03	1.25 (0.91-1.73)	0.17	1.12 (0.76-1.64)	0.57
	Postnatal								
	8 weeks [*n* = 11429]	2.65 (2.06-3.41)	<0.001	1.76 (1.34-2.32)	<0.001	1.72 (1.27-2.32)	<0.001	1.68 (1.19-2.38)	0.003
	8 months [*n* = 10923]	2.65 (2.03-3.45)	<0.001	1.80 (1.34-2.42)	<0.001	1.72 (1.25-2.37)	0.001	1.74 (1.21-2.50)	0.003
10 years	Antenatal								
	18 weeks [*n* = 11657]	2.06 (1.58-2.96)	<0.001	1.25 (0.91-1.71)	0.16	1.19 (0.85-1.66)	0.31	1.14 (0.78-1.66)	0.51
	32 weeks [*n* = 11719]	2.51 (1.96-3.21)	<0.001	1.43 (1.03-1.98)	0.03	1.41 (0.98-2.05)	0.07	1.38 (0.91-2.09)	0.13
	Postnatal								
	8 weeks [*n* = 11429]	2.33 (1.77-3.06)	<0.001	1.58 (1.18-2.13)	0.002	1.56 (1.11-2.18)	0.01	1.40 (0.96 -2.04)	0.08
	8 months [*n* = 10923]	2.49 (1.90-3.26)	<0.001	1.76 (1.31-2.38)	<0.001	1.65 (1.18-2.32)	0.003	1.57 (1.07-2.29)	0.02
13 years	Antenatal								
	18 weeks [*n* = 11657]	2.09 (1.63-2.68)	<0.001	1.29 (0.98-1.71)	0.07	1.29 (0.95-1.75)	0.10	1.19 (0.85-1.69)	0.31
	32 weeks [*n* = 11719]	2.26 (1.74-2.94)	<0.001	1.31 (0.96-1.79)	0.09	1.31 (0.92-1.86)	0.13	1.19 (0.91-1.76)	0.38
	Postnatal								

© 2021 Dachew BA et al. *JAMA Network Open*.

	8 weeks [*n* = 11429]	2.22 (1.70-2.88)	<0.001	1.52 (1/15-2.02)	0.004	1.47 (1.06-2.05)	0.02	1.39 (0.96-2.01)	0.09
	8 months [*n* = 10923]	2.10 (1.61-2.75)	<0.001	1.44 (1.07-1.94)	0.02	1.45 (1.03-2.03)	0.03	1.41 (0.95-2.01)	0.09
15 years	Antenatal								
	18 weeks [*n* = 11657]	1.87 (1.48-2.37)	<0.001	1.20 (0.93-1.54)	0.16	1.2 (0.91-1.59)	0.20	1.17 (0.86-1.58)	0.32
	32 weeks [*n* = 11719]	2.13 (1.68-2.70)	<0.001	1.29 (0.98-1.70)	0.07	1.29 (0.94-1.78)	0.12	1.24 (0.89-1.74)	0.21
	Postnatal								
	8 weeks [*n* = 11429]	1.95 (1.47-2.59)	<0.001	1.39 (1.02-1.91)	0.04	1.34 (0.95-1.89)	0.09	1.26 (0.86-1.82)	0.23
	8 months [*n* = 10923]	2.05 (1.57-2.68)	<0.001	1.51 (1.12-2.02)	0.006	1.50 (1.07-2.10)	0.02	1.46 (1.01-2.10)	0.04

Model 1 was unadjusted; Model 2 adjusted for maternal age, income, educational status, ethnicity, parity, pre-pregnancy BMI, pregnancy diabetes, urinary tract infection (UTI) during pregnancy, pre-eclampsia, alcohol drinking during pregnancy, smoking during pregnancy, maternal anxiety, offspring sex and gestational ages at delivery; Model 3 further adjusted for comorbid CD in each age group, and Model 4 additionally adjusted for comorbid ADHD in each age group.

complete exposure data and imputed missing outcome and covariates.

© 2021 Dachew BA et al. *JAMA Network Open*.

eReferences

1. Boyd A, Golding J, Macleod J, et al. Cohort Profile: the 'children of the 90s'--the index offspring of the Avon Longitudinal Study of Parents and Children. *Int J Epidemiol.* 2013;42(1):111-127.
2. Fraser A, Macdonald-Wallis C, Tilling K, et al. Cohort Profile: the Avon Longitudinal Study of Parents and Children: ALSPAC mothers cohort. *Int J Epidemiol.* 2013;42(1):97-110.
3. Golding J, Pembrey M, Jones R. ALSPAC--the Avon Longitudinal Study of Parents and Children. I. Study methodology. *Paediatr Perinat Epidemiol.* 2001;15(1):74-87.
4. Avon Longitudinal Study of Parents and Children (ALSPAC). Explore data and samples. University of Bristol, UK. http://www.bristol.ac.uk/alspac/researchers/our-data. Published 2021. Accessed 08 July, 2021.

© 2021 Dachew BA et al. *JAMA Network Open*.
